# Analysis of treatment options and survival outcomes for patients with localized prostate cancer: a focus on androgen deprivation therapy

**DOI:** 10.3389/fonc.2025.1708823

**Published:** 2025-10-31

**Authors:** Zhiqiang Li, Shiwei Sun, Chenghao Tan, Huwei Yan, Yali Zhang, Yingzhong Yang, Gengyan Xiong

**Affiliations:** ^1^ Department of Urology, Peking University First Hospital, Taiyuan Hospital, Taiyuan, China; ^2^ Department of Urology, Peking Union Medical College Hospital, Chinese Academy of Medical Sciences and Peking Union Medical College, Beijing, China; ^3^ Department of Urology, Peking University First Hospital, Beijing, China; ^4^ Department of Pathology, Peking University First Hospital, Taiyuan Hospital, Taiyuan, China

**Keywords:** androgen deprivation therapy, prostate cancer, survival, Cox proportional hazards regression, propensity score matching

## Abstract

**Background:**

Management of localized prostate cancer (PCa) remains challenging in resource-limited settings where access to surgery and radiotherapy is restricted. This study assessed the survival outcomes of patients receiving androgen deprivation therapy (ADT) alone compared with other modalities.

**Methods:**

We retrospectively analyzed patients with localized PCa treated with ADT at Taiyuan Central Hospital of Shanxi Medical University from 2002 to 2023. Cox regression identified prognostic factors for overall survival (OS), disease-specific survival (DSS), and progression-free survival (PFS). Outcomes were compared with SEER database cohorts receiving radical prostatectomy (RP), radiotherapy (RT), or no treatment. Propensity score matching (PSM) was used to balance baseline characteristics.

**Results:**

Among 86 patients in the ADT cohort, the median follow-up was 2,152.5 days. Median OS was 2,378 days, with 5-year OS, DSS, and PFS rates of 58.4%, 85.2%, and 72.5%, respectively. Cox analysis identified prostate-specific antigen, ISUP grade, and body mass index as independent predictors of PFS. After PSM for age and ISUP grade, the ADT group showed significantly better OS and DSS than RP, RT, or no treatment cohorts in the SEER database.

**Conclusions:**

ADT demonstrated favorable survival outcomes compared with RP and RT in elderly patients with high-grade localized PCa. These results highlight ADT as a potential alternative where invasive options are less feasible, providing insights into optimizing treatment strategies for resource-limited settings.

## 1 Introduction

Prostate cancer (PCa) is the second most common malignancy in men globally. According to data from the International Agency for Research on Cancer (IARC), the annual incidence of prostate cancer is rising worldwide, with a particularly high prevalence in developed countries (1). Although the incidence of PCa is relatively low in developing countries, the rate of diagnosis is increasing owing to lifestyle changes and advancements in medical technology (2, 3).

The most commonly used pathological grading systems for PCa are the Gleason score and the International Society of Urological Pathology (ISUP) grade (4). Staging is typically performed using the tumor, node, and metastasis (TNM) classification system, which categorizes PCa into three primary stages based on tumor progression: localized (T_1-2_N_0_M_0_), locally advanced (T_3-4_N_0_M_0_ or T_X_N_1_M_0_), and metastatic (T_X_N_X_M_1_) (5). Radical prostatectomy (RP) is generally recommended as the primary treatment for localized PCa (6). However, in developing countries, limited healthcare resources and economic constraints pose significant challenges for treatment choices (7). Consequently, androgen deprivation therapy (ADT) has been becoming a widely used treatment modality in these regions (8, 9).

Despite its extensive use, there is a relative scarcity of comparative studies on the efficacy and survival outcomes of ADT in patients with localized PCa in developing countries (8). This study aimed to analyze the survival outcomes of patients with localized PCa who underwent ADT at Taiyuan Central Hospital of Shanxi Medical University (Peking University First Hospital Taiyuan Hospital) from June 2002 to August 2023. This analysis will be contrasted with treatment data from the Surveillance, Epidemiology, and End Results Program (SEER) database, which includes a large cohort of patients from the United States. The objective of this study was to evaluate the efficacy, safety, and survival benefits of ADT in these specific patient populations and to provide a scientific basis for treatment strategies.

## 2 Methods

### 2.1 General data

This study retrospectively collected data from patients diagnosed with primary PCa who were treated with ADT at Taiyuan Central Hospital of Shanxi Medical University between June 2002 and August 2023, with follow-up periods exceeding one year, forming the ADT cohort. The inclusion criteria were as follows:

1. diagnosis of PCa based on biopsy, or postoperative pathology after transurethral resection of the prostate (TURP)
2. confirmation of localized PCa through radiological examination, such as
3. Positron Emission Tomography-Computed Tomography (PET-CT)
4. patients who underwent regular ADT
5. complete medical records; and
6. comprehensive follow-up data.

Exclusion criteria included:

1. patients diagnosed with other types of prostate tumors,
2. unclear Gleason score in pathology,
3. evidence of distant metastasis on radiological examination,
4. receiving other treatments such as RP, chemotherapy, radiotherapy (RT), or other therapies;
5. patients who refused ADT or discontinued treatment prematurely; and
6. loss to follow-up or follow-up duration of less than one year. Data from the SEER database for patients with primary localized PCa (ICD-O: 8140/3, PRIMARY SITE = C61.9) who received other treatments or no treatment were used as control cohorts. To ensure temporal consistency across cohorts, we specified the study periods for each group. The ADT cohort from our institution included patients diagnosed between 2002 and 2023, whereas the SEER database initially comprised more than one million cases from 2000 to 2021. After applying inclusion and exclusion criteria, the final analytical cohort contained 293,397 patients diagnosed between 2010 and 2021. Propensity score matching further yielded 258 patients (86 in each of the RP, RT, and non-treatment groups) within the same 2010–2021 timeframe. This period corresponds to the era following the widespread adoption of robotic-assisted radical prostatectomy, ensuring that surgical outcomes reflect modern operative techniques.

The inclusion and exclusion criteria were similar to those of the ADT cohort: Inclusion criteria were.

1. primary localized PCa;
2. complete treatment information;
3. comprehensive follow-up data.

The exclusion criteria were.

**Figure 1:**
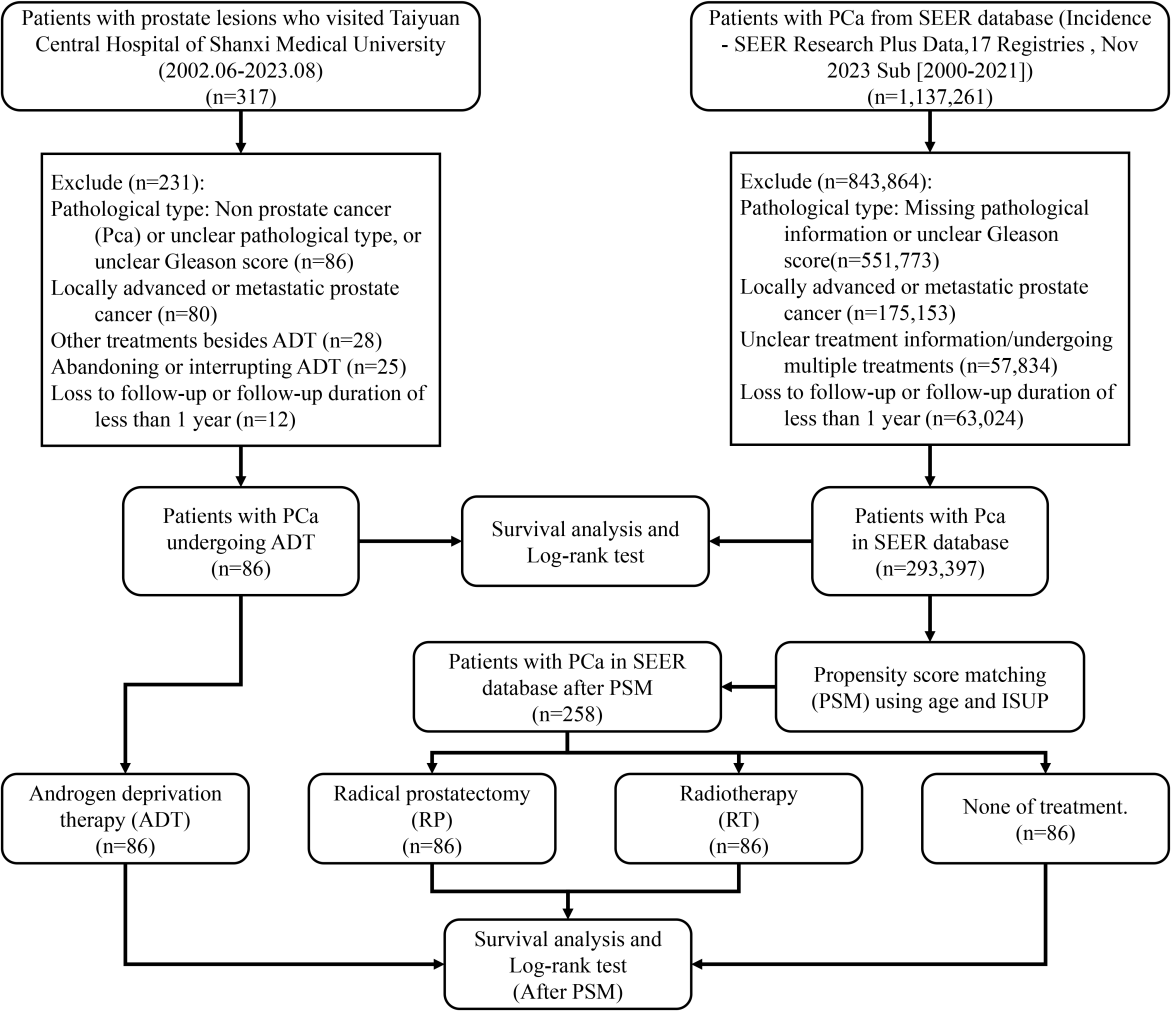
Study flowchart.

1. non-primary prostate tumors,
2. incomplete pathological information or unclear Gleason score,
3. locally advanced or metastatic PCa,
4. incomplete treatment information or concurrent multiple therapies, and
5. loss to follow-up or follow-up duration of less than one year (**Figure 1**).

### 2.2 Research methods

This study analyzed the survival outcomes of patients with malignant prostate tumors based on their baseline characteristics, laboratory results, pathological findings, and treatment regimens. Key factors included age, PSA, fPSA, Gleason score, ISUP grade, BMI, previous history (such as hypertension, diabetes mellitus, coronary artery disease, and cerebral infarction), daily habits (such as current smoking and drinking), and ADT details. ADT modalities encompassed bilateral orchiectomy, Luteinizing Hormone-Releasing Hormone agonists (LHRHa) (Leuprorelin, Goserelin), and Non-Steroidal Anti-Androgen (NSAA) (Bicalutamide, Apalutamide, Enzalutamide, Darolutamide).

Overall survival (OS) was defined as the period from diagnosis to death or the last follow-up, with death as the event. Disease-specific survival (DSS) was defined as the period from diagnosis to death due to PCa or last follow-up. Progression-free survival (PFS) was defined as the interval from the start of treatment to disease progression (including radiographic or PSA progression, advancing to castration-resistant prostate cancer) or death.

### 2.3 Statistical methods

Data analysis was conducted using R 4.3.2 (R Foundation for Statistical Computing, Vienna, Austria). Continuous variables following a normal distribution were expressed as mean ± SD and compared between groups using an independent sample t-test; non-normally distributed continuous variables were presented as median (interquartile range) and compared using the rank-sum test. Categorical variables were expressed as frequencies and percentages (%) and compared using Pearson’s χ2 test or Fisher’s exact test, depending on the minimum expected cell counts. The “survival” package was used for univariate Cox proportional hazards regression analysis to identify factors affecting OS, DSS, and PFS in patients undergoing ADT. Kaplan–Meier (KM) curves and Log-rank tests were used to compare OS and DSS between different treatments. Propensity score matching (PSM) was conducted using the “MatchIt” package based on age and ISUP grade, followed by KM curve comparison and Log-rank tests to more accurately evaluate differences in OS and DSS between different treatments. Statistical significance was set at P < 0.05.

## 3 Results

### 3.1 General characteristics

A total of 86 patients who underwent ADT were included in this study, all of whom completed the full follow-up. The median follow-up duration was 2,152.5 days [1,176.75, 3,540.25], with the longest follow-up duration lasting 5,045 days. The baseline characteristics of the patients are summarized in **Table 1**. At the time of initial diagnosis, the median age was 76.00 years. The distribution of ISUP grades was as follows: Grade 1 in 22 patients (25.6%), Grade 2 in 21 patients (24.4%), Grade 3 in 9 patients (10.5%), Grade 4 in 17 patients (19.8%), and Grade 5 in 17 patients (19.8%). All patients received one or more forms of ADT, with 73 patients (84.9%) receiving NSAA therapy (including Bicalutamide, Apalutamide, Enzalutamide and Darolutamide), and 66 patients (76.7%) opted for LHRHa therapy (including Leuprorelin and Goserelin). In addition, 20 patients (23.3%) underwent bilateral orchiectomy.

**Table 1:** Baseline characteristics of ADT cohort.

Variables	Total cohort (n=86)	Overall survival		t/Z/χ^2^	P	Disease-specific survival		t/Z/χ^2^	P
		Dead (n=43)	Alive (n=43)			Died due to PCa (n=9)	Others (n=77)		
Age	76.0[72.0,80.0]	78.0[72.5,80.5]	76.0[71.0,80.0]	1.36	0.175	74.0[72.0,78.0]	77.0[72.0,80.0]	0.712	0.48
BMI	23.64(± 3.75)	23.40(± 4.18)	23.88(± 3.30)	0.584	0.561	23.13(± 3.58)	23.70(± 3.79)	0.428	0.67
PSA	35.40[12.90,81.28]	49.40[18.30,83.44]	23.57[8.33,60.06]	1.645	0.101	83.5[67.03,107.0]	29.30[12.30,59.60]	1.905	0.058
fPSA	4.21[1.93,14.61]	8.79[2.79,19.90]	4.21[1.60,10.36]	1.188	0.237	13.44[9.84,21.80]	4.11[1.74,13.25]	2.271	0.024
Gleason				4.445	0.617			7.09	0.313
3 + 3	22(25.6)	12(54.5)	10(45.5)			1(4.5)	21(95.5)		
3 + 4	21(24.4)	11(52.4)	10(47.6)			1(4.8)	20(95.2)		
4 + 3	9(10.5)	6(66.7)	3(33.3)			1(11.1)	8(88.9)		
4 + 4	17(19.8)	5(29.4)	12(70.6)			2(11.8)	15(88.2)		
4 + 5	12(14.0)	6(50.0)	6(50.0)			2(16.7)	10(83.3)		
5 + 4	2(2.3)	1(50.0)	1(50.0)			1(50.0)	1(50.0)		
5 + 5	3(3.5)	2(66.7)	1(33.3)			1(33.3)	2(66.7)		
ISUP				4.171	0.383			4.683	0.321
1	22(25.6)	12(54.5)	10(45.5)			1(4.5)	21(95.5)		
2	21(24.4)	11(52.4)	10(47.6)			1(4.8)	20(95.2)		
3	9(10.5)	6(66.7)	3(33.3)			1(11.1)	8(88.9)		
4	17(19.8)	5(29.4)	12(70.6)			2(11.8)	15(88.2)		
5	17(19.8)	9(52.9)	8(47.1)			4(23.5)	13(76.5)		
Orchiectomy				16.679	<0.001			0.115	0.734
No	66(76.7)	25(37.9)	41(62.1)			6(9.1)	60(90.9)		
Yes	20(23.3)	18(90.0)	2(10.0)			3(15.0)	17(85.0)		
NSAA				7.34	0.007			0.001	0.999
No	13(15.1)	11(84.6)	2(15.4)			1(7.7)	12(92.3)		
Yes	73(84.9)	32(43.8)	41(56.2)			8(11.0)	65(89.0)		
LHRHα				9.382	0.002			0.001	0.999
No	20(23.3)	16(80.0)	4(20.0)			2(10.0)	18(90.0)		
Yes	66(76.7)	27(40.9)	39(59.1)			7(10.6)	59(89.4)		
Hypertension				0.047	0.829			0.001	0.999
No	45(52.3)	22(48.9)	23(51.1)			5(11.1)	40(88.9)		
Yes	41(47.7)	21(51.2)	20(48.8)			4(9.8)	37(90.2)		
Diabetes mellitus				1.229	0.268			1.13	0.288
No	70(81.4)	37(52.9)	33(47.1)			9(12.9)	61(87.1)		
Yes	16(18.6)	6(37.5)	10(62.5)			0(0.0)	16(100.0)		
Coronary artery disease				0.104	0.747			0.472	0.492
No	75(87.2)	37(49.3)	38(50.7)			9(12.0)	66(88.0)		
Yes	11(12.8)	6(54.5)	5(45.5)			0(0.0)	11(100.0)		
Cerebral infarction				0.081	0.776			0.001	0.999
No	71(82.6)	35(49.3)	36(50.7)			7(9.9)	64(90.1)		
Yes	15(17.4)	8(53.3)	7(46.7)			2(13.3)	13(86.7)		
Current smoker				0.049	0.825			0.001	0.999
No	53(61.6)	26(49.1)	27(50.9)			6(11.3)	47(88.7)		
Yes	33(38.4)	17(51.5)	16(48.5)			3(9.1)	30(90.9)		
Current drinker				0.001	0.999			0.42	0.517
No	64(74.4)	32(50.0)	32(50.0)			8(12.5)	56(87.5)		
Yes	22(25.6)	11(50.0)	11(50.0)			1(4.5)	21(95.5)		

Data are n (%) or median [IQR].

The median OS was 2,378.0 days, with 1-, 3-, 5-, and 10-year OS rates of 93.00%, 76.00%, 58.40%, and 27.30%, respectively. The DSS rates at 1, 3, 5, and 10 years were 98.80%, 95.70%, 85.20%, and 76.70%, respectively. The PFS rates at 1, 3, 5, and 10 years were 93.90%, 83.40%, 72.50%, and 65.00%, respectively.

### 3.2 Univariate Cox proportional-hazards regression analysis

**Figure 2:**
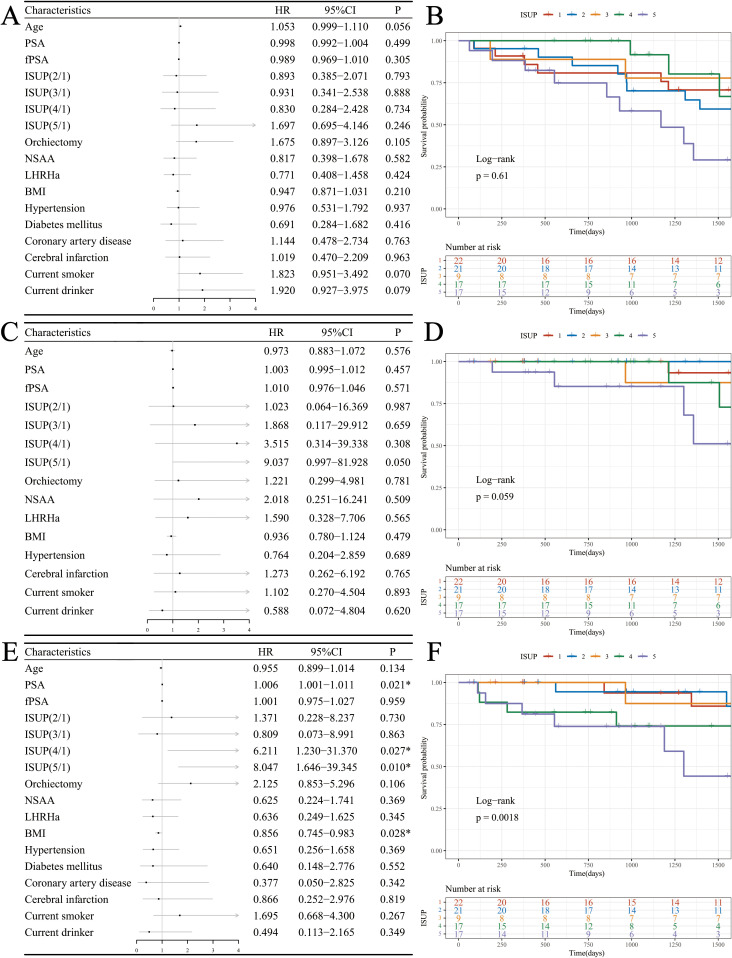
Survival analysis in the ADT cohort. **(A)** Forest plot of OS; **(B)** KM curves of OS based on ISUP grade; **(C)** Forest plot of DSS; **(D)** KM curves of DSS based on ISUP grade; **(E)** Forest plot of PFS; **(F)** KM curves of PFS based on ISUP grade.

Univariate Cox proportional hazards regression analysis indicated that none of the factors significantly affected the OS of patients receiving ADT. However, age (HR: 1.053, 95% CI: 0.999-1.110, P = 0.056), current smoking status (HR: 1.823, 95% CI: 0.951-3.492, P = 0.070), and current alcohol status (HR: 1.920, 95% CI: 0.927-3.975, P = 0.079) exhibited potential influences on OS (**Figures 2A, B**). While none of the factors showed statistically significant effects on DSS, the ISUP grade (HR: 9.037, 95% CI: 0.997-81.928, P = 0.050) demonstrated a slight impact (**Figures 2C,D**). The following factors were found to influence PFS: PSA (HR: 1.006, 95% CI: 1.001-1.011, P = 0.021), ISUP grade (HR: 8.047, 95% CI: 1.646-39.345, P = 0.010), and body mass index (BMI) (HR: 0.856, 95% CI: 0.745-0.983, P = 0.028) (**Figures 2E, F**).

### 3.3 Comparison of treatment modalities

**Figure 3:**
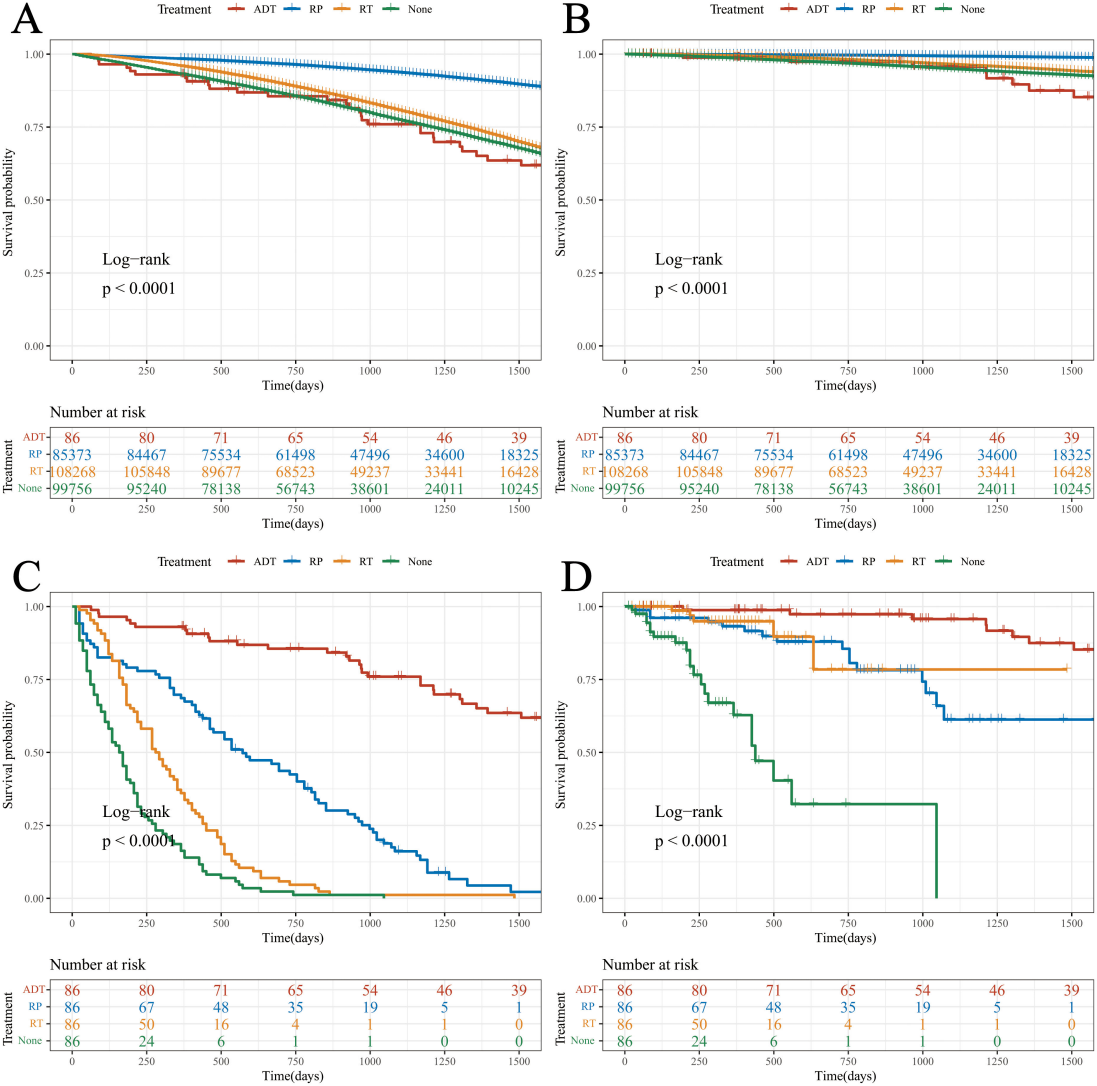
Survival analysis of the different cohorts. **(A)** OS (before PSM); **(B)** DSS (before PSM); **(C)** OS (after PSM); **(D)** DSS (after PSM).

Data were extracted from the SEER database, including patients with localized PCa who underwent RP or RT, and those who did not receive any treatment. These data were compared with those a of cohort of patients treated with ADT in this study (**Table 2**). The Log-rank test revealed that the OS of patients treated with RP significantly surpassed that of the other three cohorts (**Figures 3A, B**).

**Table 2:** Baseline characteristics of SEER database.

Variables	Total cohort in SEER (n=294514)	Total cohort (after PSM) (n=258)	Classified by treatment (after PSM)		
			RP (n=86)	RT (n=86)	None (n=86)
Age	66.0[60.0,71.0]	75.0[70.0,80.0]	76.0[71.3,80.0]	76.0[70.0,80.0]	73.0[65.5,80.0]
Gleason					
3 + 3	118789(40.5)	66(25.6)	22(25.6)	22(25.6)	22(25.6)
3 + 4	94869(32.3)	63(24.4)	21(24.4)	21(24.4)	21(24.4)
4 + 3	1705(0.6)	1(0.4)	1(1.2)	0(0.0)	0(0.0)
3 + 5	39839(13.6)	27(10.5)	9(10.5)	9(10.5)	9(10.5)
4 + 4	21221(7.2)	49(19.0)	15(17.4)	17(19.8)	17(19.8)
5 + 3	9407(3.2)	15(5.8)	12(14.0)	2(2.3)	1(1.2)
4 + 5	436(0.1)	1(0.4)	1(1.2)	0(0.0)	0(0.0)
5 + 4	6141(2.1)	36(14.0)	5(5.8)	15(17.4)	16(18.6)
5 + 5	990(0.3)	0(0.0)	0(0.0)	0(0.0)	0(0.0)
ISUP					
1	118789(40.5)	66(25.6)	22(25.6)	22(25.6)	22(25.6)
2	94869(32.3)	63(24.4)	21(24.4)	21(24.4)	21(24.4)
3	39839(13.6)	27(10.5)	9(10.5)	9(10.5)	9(10.5)
4	23362(8.0)	51(19.8)	17(19.8)	17(19.8)	17(19.8)
5	16538(5.6)	51(19.8)	17(19.8)	17(19.8)	17(19.8)
PSA	6.4[4.8,9.6]	8.4[5.6,15.4]	6.3[3.2,8.6]	9.8[6.7,15.9]	10.2[7.1,26.7]
T stage					
1a	5506(1.9)	7(2.7)	1(1.2)	2(2.3)	4(4.7)
1b	1692(0.6)	3(1.2)	1(1.2)	0(0.0)	2(2.3)
1c	143514(48.9)	90(34.9)	1(1.2)	41(47.7)	48(55.8)
2a	45569(15.5)	83(32.2)	24(27.9)	31(36.0)	28(32.6)
2b	32568(11.1)	21(8.1)	12(14.0)	8(9.3)	1(1.2)
2c	64548(22.0)	54(20.9)	47(54.7)	4(4.7)	3(3.5)
Surgery					
No	208024(70.9)	172(66.7)	0(0.0)	86(100.0)	86(100.0)
Yes	85373(29.1)	86(33.3)	86(100.0)	0(0.0)	0(0.0)
Radiotherapy					
No	185129(63.1)	172(66.7)	86(100.0)	0(0.0)	86(100.0)
Yes	108268(36.9)	86(33.3)	0(0.0)	86(100.0)	0(0.0)
Residence					
Rural	34350(11.7)	72(27.9)	15(17.4)	28(32.6)	29(33.7)
Urban	259047(88.3)	186(72.1)	71(82.6)	58(67.4)	57(66.3)

Data are n (%) or median [IQR].

Notably, there were significant differences in age and ISUP grade between the ADT cohort and the other three cohorts. To enhance the scientific rigor of the statistics, PSM was performed based on the age and ISUP grade.

Following PSM, Log-rank tests indicated that both OS and DSS in the ADT cohort were superior to those in the other three groups. This suggests that, under comparable age and pathological stage conditions, the therapeutic efficacy of ADT appears to be greater than that of RP and RT (**Figures 3C, D**).

## 4 Discussion

PCa is the most common malignancy of the male genitourinary system. According to the World Health Organization’s GLOBOCAN 2020 statistics, PCa is the second most prevalent cancer among men worldwide, after lung cancer (1, 10). The incidence of PCa varies significantly by region and ethnicity, with rates in developed countries being three times higher than in developing countries (11–13). China, a typical developing nation, has traditionally had lower incidence and mortality rates of PCa (14). However, in recent years, there has been a notable increase in these rates owing to the widespread adoption of screening and advances in medical technology (15, 16).

Surgery is the preferred treatment for non-metastatic PCa, with RP being highly recommended for localized cases (6, 17). Additionally, the proportion of patients opting for RT, such as permanent seed implantation, has been steadily increasing (18). Given that PCa cells are highly sensitive to androgens, which regulate cell proliferation and survival through androgen receptors, ADT has garnered increasing attention as a potential treatment modality for PCa (19).

However, ADT is rarely recommended as a standalone treatment for localized PCa. It is typically used as an adjunct to RT and salvage RT, or as a treatment for locally advanced or metastatic PCa (20–23). In developing countries, where economic and healthcare resources are limited, patients and their families may have reservations about invasive treatments, such as surgery (16). Additionally, patients in these regions are often diagnosed at an older age with higher Gleason scores and ISUP grades, which reduces their tolerance to surgical interventions. Consequently, elderly patients with localized PCa may consider ADT an alternative treatment option.

This study analyzed the survival outcomes of patients with localized PCa treated exclusively with ADT. The results showed no statistically significant impact of the various factors on OS or DSS. However, certain adverse lifestyle factors, such as smoking and alcohol consumption, may potentially influence OS. Furthermore, PSA level, ISUP grade, and BMI were found to have a significant impact on PFS.

PSA is a protein secreted by prostate epithelial cells, and its levels increase as PCa progresses, particularly with higher Gleason grades, leading to the release of more PSA into the bloodstream (24). Therefore, patients with elevated PSA levels and ISUP grades generally have poorer prognoses (25). When BMI is within the normal range, a higher BMI indicates better nutritional reserves, which may enhance the patient’s ability to tolerate the challenges of PCa (26). The G8 screening tool, which is widely used for predicting PCa survival, also identifies a BMI >23 as a favorable prognostic indicator, while a BMI <19 is considered indicative of poor prognosis (27). The influence of adverse lifestyle factors on cancer prognosis has been well-documented in the literature (28–30).

The study also revealed that patients in the ADT cohort from China were significantly older at initial diagnosis than those in the SEER database (76.00 [72.00, 80.00] vs. 66.00 [60.00, 71.00], Z = 10.096, P<0.001), with higher Gleason scores and ISUP grades (only 50.0% of the Chinese ADT cohort were classified as ISUP ≤2 compared with 72.8% in the SEER database; χ²=52.472, P<0.001). To ensure the scientific rigor of these comparisons, PSM was performed according to age and ISUP grade (31). After PSM, patients in the ADT cohort demonstrated significantly better OS and DSS than those who underwent RP, RT, or received no treatment. It should be noted that the seemingly superior DSS observed in the ADT cohort may be subject to several confounding influences. First, selection bias is inherent in retrospective designs: most patients treated with ADT were elderly with multiple comorbidities and were more likely to die from non–cancer-related causes, leading to an apparent improvement in DSS. Second, patients undergoing RP or RT in the SEER database typically received more extensive staging examinations, introducing possible stage migration. Third, the SEER registry does not consistently capture details of adjuvant or salvage treatments, such as postoperative or post-radiation ADT, which might underestimate true survival outcomes in these groups. Moreover, differences in follow-up duration and data completeness between institutional and SEER cohorts may have introduced residual bias. Therefore, the observed DSS advantage with ADT should be interpreted with caution. This improvement may be attributed to poorer tolerance in older patients with higher ISUP grades, as well as an increased risk of mortality from non-PCa-related diseases (32). Previous guidelines have recommended a quality-of-life-focused approach with symptom-oriented treatment, such as watchful waiting (WW), particularly for patients with limited surgical tolerance (17). However, as economic conditions improve and life expectancy increases, some patients may perceive WW as a form of treatment abandonment, leading to a growing preference for ADT. This study further confirms the feasibility of ADT as a standalone treatment option for localized PCa.

The limitations of this study include the use of the ADT cohort from a single center in China, compared with data from the SEER database representing U.S. patients, which may introduce some discrepancies. To minimize these differences, PSM for age and ISUP grade was performed before the comparison.

## 5 Conclusions

This study confirmed that the primary factors influencing PFS in patients treated with ADT were the PSA level, ISUP grade, and BMI. Survival analysis adjusted for age and ISUP through PSM demonstrated that ADT provides superior outcomes compared to RP and RT for elderly patients with high-stage localized PCa, offering better survival rates. These findings are significant for understanding the treatment options for elderly patients with localized PCa and provide valuable insights for PCa treatment strategies. By thoroughly analyzing the efficacy and survival outcomes of ADT, we aimed to improve the clinical prognosis and quality of life of these patients.

## Data Availability

The original contributions presented in the study are included in the article/**Supplementary Material**. Further inquiries can be directed to the corresponding authors.

## References

[1] Sung H Ferlay J Siegel RL Laversanne M Soerjomataram I Jemal A. Global cancer statistics 2020: GLOBOCAN estimates of incidence and mortality worldwide for 36 cancers in 185 countries. CA Cancer J Clin. (2021) 71:209–49. doi: 10.3322/caac.21660, PMID: 33538338

[2] Ha Chung B Horie S Chiong E. The incidence, mortality, and risk factors of prostate cancer in Asian men. Prost Int. (2019) 7:1–8. doi: 10.1016/j.prnil.2018.11.001, PMID: 30937291 PMC6424686

[3] Chen R Ren S Yiu MK Fai NC Cheng WS Ian LH. Prostate cancer in Asia: A collaborative report. Asian J Urol. (2014) 1:15–29. doi: 10.1016/j.ajur.2014.08.007, PMID: 29511634 PMC5832886

[4] Epstein JI Egevad L Amin MB Delahunt B Srigley JR Humphrey PA. The 2014 international society of urological pathology (ISUP) consensus conference on gleason grading of prostatic carcinoma: definition of grading patterns and proposal for a new grading system. Am J Surg Pathol. (2016) 40:244–52. doi: 10.1097/PAS.0000000000000530, PMID: 26492179

[5] Paner GP Stadler WM Hansel DE Montironi R Lin DW Amin MB. Updates in the eighth edition of the tumor-node-metastasis staging classification for urologic cancers. Eur Urol. (2018) 73:560–9. doi: 10.1016/j.eururo.2017.12.018, PMID: 29325693

[6] Milonas D Giesen A Laenen A Devos G Briganti A Gontero P. Effect of radical prostatectomy on survival for men with high-risk nonmetastatic prostate cancer features selected according to STAMPEDE criteria: an EMPaCT study. Eur Urol Oncol. (2024) 7:1478–1486. doi: 10.1016/j.euo.2024.05.016, PMID: 38997858

[7] Collaborators GDaI. Global incidence, prevalence, years lived with disability (YLDs), disability-adjusted life-years (DALYs), and healthy life expectancy (HALE) for 371 diseases and injuries in 204 countries and territories and 811 subnational locations, 1990-2021: a systematic analysis for the Global Burden of Disease Study 2021. Lancet.(2024) 403:2133–61. doi: 10.1016/S0140-6736(24)00757-8, PMID: 38642570 PMC11122111

[8] Hussain M Fizazi K Shore ND Heidegger I Smith MR Tombal B. Metastatic hormone-sensitive prostate cancer and combination treatment outcomes: A review. JAMA Oncol. (2024) 10:807–20. doi: 10.1001/jamaoncol.2024.0591, PMID: 38722620

[9] Konoshenko MY Bryzgunova OE Laktionov PP. miRNAs and androgen deprivation therapy for prostate cancer. Biochim Biophys Acta Rev Cancer. (2021) 1876:188625. doi: 10.1016/j.bbcan.2021.188625, PMID: 34534639

[10] Netto GJ Amin MB Berney DM Compérat EM Gill AJ Hartmann A. The 2022 world health organization classification of tumors of the urinary system and male genital organs-part B: prostate and urinary tract tumors. Eur Urol. (2022) 82:469–82. doi: 10.1016/j.eururo.2022.07.002, PMID: 35965208

[11] De Angelis R Demuru E Baili P Troussard X Katalinic A Chirlaque Lopez MD. Complete cancer prevalence in Europe in 2020 by disease duration and country (EUROCARE-6): a population-based study. Lancet Oncol. (2024) 25:293–307. doi: 10.1016/S1470-2045(23)00646-0, PMID: 38307102

[12] Sivakumar S Lee JK Moore JA Hopkins J Newberg JY Madison R. Comprehensive genomic profiling and treatment patterns across ancestries in advanced prostate cancer: a large-scale retrospective analysis. Lancet Dig Health. (2023) 5:e380–e9. doi: 10.1016/S2589-7500(23)00053-5, PMID: 37236698

[13] Schafer EJ Jemal A Wiese D Sung H Kratzer TB Islami F. Disparities and trends in genitourinary cancer incidence and mortality in the USA. Eur Urol. (2023) 84:117–26. doi: 10.1016/j.eururo.2022.11.023, PMID: 36566154

[14] Chen W Zheng R Baade PD Zhang S Zeng H Bray F. Cancer statistics in China, 2015. CA Cancer J Clin. (2016) 66:115–32. doi: 10.3322/caac.21338, PMID: 26808342

[15] Zheng R Zhang S Zeng H Wang S Sun K Chen R. Cancer incidence and mortality in China, 2016. J Natl Cancer Cent. (2022) 2:1–9. doi: 10.1016/j.jncc.2022.02.002, PMID: 39035212 PMC11256658

[16] Soerjomataram I Cabasag C Bardot A Fidler-Benaoudia MM Miranda-Filho A Ferlay J. Cancer survival in Africa, central and south America, and Asia (SURVCAN-3): a population-based benchmarking study in 32 countries. Lancet Oncol. (2023) 24:22–32. doi: 10.1016/S1470-2045(22)00704-5, PMID: 36603919

[17] Bill-Axelson A Holmberg L Garmo H Taari K Busch C Nordling S. Radical prostatectomy or watchful waiting in prostate cancer - 29-year follow-up. N Engl J Med. (2018) 379:2319–29. doi: 10.1056/NEJMoa1807801, PMID: 30575473

[18] Wallis CJD Glaser A Hu JC Huland H Lawrentschuk N Moon D. Survival and complications following surgery and radiation for localized prostate cancer: an international collaborative review. Eur Urol. (2018) 73:11–20. doi: 10.1016/j.eururo.2017.05.055, PMID: 28610779

[19] Wu S Li K Zhang Y Wang L Zhu B Wang W. Men’s symptom experience throughout androgen deprivation therapy for prostate cancer: A systematic review and meta-aggregation. Int J Nurs Stud. (2024) 157:104796. doi: 10.1016/j.ijnurstu.2024.104796, PMID: 38824718

[20] Supiot S Vaugier L Pasquier D Buthaud X Magné N Peiffert D. OLIGOPELVIS GETUG P07, a multicenter phase II trial of combined high-dose salvage radiotherapy and hormone therapy in oligorecurrent pelvic node relapses in prostate cancer. Eur Urol. (2021) 80:405–14. doi: 10.1016/j.eururo.2021.06.010, PMID: 34247896

[21] Wang C Raldow AC Nickols NG Nguyen PL Spratt DE Dess RT. Underutilization of androgen deprivation therapy with external beam radiotherapy in men with high-grade prostate cancer. Eur Urol Oncol. (2021) 4:327–30. doi: 10.1016/j.euo.2019.01.006, PMID: 31411981

[22] Jones C Gray S Brown M Brown J McCloskey E Rai BP. Risk of fractures and falls in men with advanced or metastatic prostate cancer receiving androgen deprivation therapy and treated with novel androgen receptor signalling inhibitors: A systematic review and meta-analysis of randomised controlled trials. Eur Urol Oncol. (2024) 7:993–1004. doi: 10.1016/j.euo.2024.01.016, PMID: 38383277

[23] Gandaglia G Fossati N Montorsi F Briganti A. Does radiotherapy plus androgen-deprivation therapy represent the best treatment approach in elderly patients with locally advanced prostate cancer? J Clin Oncol. (2015) 33:2831–2. doi: 10.1200/JCO.2015.61.0964, PMID: 26215938

[24] Bakht MK Yamada Y Ku S-Y Venkadakrishnan VB Korsen JA Kalidindi TM. Landscape of prostate-specific membrane antigen heterogeneity and regulation in AR-positive and AR-negative metastatic prostate cancer. Nat Cancer. (2023) 4:699–715. doi: 10.1038/s43018-023-00539-6, PMID: 37038004 PMC10867901

[25] Bögemann M Shore ND Smith MR Tammela TLJ Ulys A Vjaters E. Efficacy and safety of darolutamide in patients with nonmetastatic castration-resistant prostate cancer stratified by prostate-specific antigen doubling time: planned subgroup analysis of the phase 3 ARAMIS trial. Eur Urol. (2023) 83:212–21. doi: 10.1016/j.eururo.2022.07.018, PMID: 36089529

[26] Hamaker ME Oosterlaan F van Huis LH Thielen N Vondeling A van den Bos F. Nutritional status and interventions for patients with cancer - A systematic review. J Geriatr Oncol. (2021) 12:6–21. doi: 10.1016/j.jgo.2020.06.020, PMID: 32616384

[27] Droz J-P Albrand G Gillessen S Hughes S Mottet N Oudard S. Management of prostate cancer in elderly patients: recommendations of a task force of the international society of geriatric oncology. Eur Urol. (2017) 72:521–31. doi: 10.1016/j.eururo.2016.12.025, PMID: 28089304

[28] Peisch SF Van Blarigan EL Chan JM Stampfer MJ Kenfield SA. Prostate cancer progression and mortality: a review of diet and lifestyle factors. World J Urol. (2017) 35:867–74. doi: 10.1007/s00345-016-1914-3, PMID: 27518576 PMC5472048

[29] Pernar CH Ebot EM Wilson KM Mucci LA. The epidemiology of prostate cancer. Cold Spring Harb Perspect Med. (2018) 8:a030361. doi: 10.1101/cshperspect.a030361, PMID: 29311132 PMC6280714

[30] Macke AJ Petrosyan A. Alcohol and prostate cancer: time to draw conclusions. Biomolecules.(2022) 12:375. doi: 10.3390/biom12030375, PMID: 35327568 PMC8945566

[31] Cook MB Hurwitz LM Geczik AM Butler EN. An up-to-date assessment of US prostate cancer incidence rates by stage and race: A novel approach combining multiple imputation with age and delay adjustment. Eur Urol. (2021) 79:33–41. doi: 10.1016/j.eururo.2020.09.041, PMID: 33092896 PMC8853735

[32] Mungovan SF Carlsson SV Gass GC Graham PL Sandhu JS Akin O. Preoperative exercise interventions to optimize continence outcomes following radical prostatectomy. Nat Rev Urol. (2021) 18:259–81. doi: 10.1038/s41585-021-00445-5, PMID: 33833445 PMC8030653

